# Efficacy and safety of dapsone as second line therapy for adult immune thrombocytopenia: A retrospective study of 42 patients

**DOI:** 10.1371/journal.pone.0187296

**Published:** 2017-10-30

**Authors:** Clémentine Estève, Maxime Samson, Alexandre Guilhem, Barbara Nicolas, Vanessa Leguy-Seguin, Sabine Berthier, Bernard Bonnotte, Sylvain Audia

**Affiliations:** Université de Bourgogne Franche Comté, CHU Dijon Bourgogne, Service de Médecine Interne et Immunologie Clinique, Centre de Référence Constitutif des Cytopénies Auto-immunes de l’adulte, Dijon, France; Keio University, JAPAN

## Abstract

Dapsone is recommended as a second line therapy in immune thrombocytopenia (ITP), but is underused because of its potential side effects. The medical charts of 42 ITP patients treated with dapsone (100 mg/day) were retrospectively reviewed in order to assess its efficacy and safety in daily clinical practice. The overall response rate was 54.8% (n = 22, with a complete response in 38.1%) with a median time to response of 29 days (24–41 days). Patients with complete response had shorter disease duration whereas no difference was observed between responders and non-responders regarding age, sex or previous treatments received. Importantly, after dapsone withdrawal, a sustained response was observed in 5 patients, representing 12% of the whole cohort. Twenty percent of patients (n = 8) relapsed on therapy after 8.1 (6.5–13.6) months. Side effects occurred in 31% (n = 13) of patients, and required dapsone withdrawal in 22% (n = 9) or dosage reduction in 10% (n = 4) of the cases. Side effects resolved in all but one case. Overall, these data support dapsone as an interesting second line therapy in ITP, with a good safety and efficacy profile at a low cost.

## Introduction

Immune thrombocytopenia (ITP) is an autoimmune disorder leading to a low platelet count responsible for bleedings of variable severity. Treatments are recommended in case of bleeding symptoms and/or platelet count below 20x10^9^/L. Steroids are used as first-line therapy, while intravenous immunoglobulins (IVIg) should be restricted to patients with severe bleeding symptoms [1–3]. Both steroids and IVIg offer high response rates, but relapses are common [1,2]. Rituximab is used as a second-line therapy with a response rate of 40% after one year follow-up, but only 20% after five years [4–6]. Of note, rituximab is expensive and could favour infections [7]. Hydroxychloroquine is usually used when antinuclear antibodies are positive, with a response rate of 60% [8]. Because of its side-effects, particularly virilization and liver cytolysis, danazol is less and less used [9,10]. Splenectomy is considered for chronic ITP, as it is the unique curative treatment with a long term response in 66–88% of patients, of whom only 15% will relapse [11,12]. Of note, long term infectious susceptibility following splenectomy requires prophylactic measures and patient education. Thrombopoietin receptor agonists (TPO-RA) have a response rate of 80%, with a suspensive effect, thus requiring long term treatment [13,14], although long-term response following their transient use have been observed in 15–30% [15–18]. Immunosuppressive drugs such as azathioprine, ciclosporine, cyclophosphamide or mycophenolate mofetil are dedicated to multirefractory patients [19].

Dapsone efficiency was first reported in the 90’s in ITP [20,21]. Since then, several studies confirmed its potential interest as second line therapy in ITP, with response rate ranging between 40–62% [10,22–26]. The mechanisms of action of dapsone remain unclear, but it has been postulated that haemolysis induced by dapsone might limit the phagocytosis of opsonized-platelets by diverting splenic macrophages [20,23].

We aimed to assess the efficacy and safety of dapsone in a retrospective monocentric study in adult ITP.

## Materials and methods

### Patients

Medical records of all patients registered in Dijon University Hospital Centre between January 2006 and August 2016 for thrombocytopenia according to the diagnosis-related group (DRG) medical information system (PMSI) were retrospectively reviewed. The study was approved by the institutional review board of the University Hospital of Dijon and the local ethics committee (*Comité de Protection des Personnes Est I*), who waived the requirement for informed consent.

Patients with a diagnosis of ITP and treated with dapsone were selected. Demographic data (age, sex, weight, bleeding score [27] at dapsone introduction), dapsone-related side effects, previous treatments and their response were collected. The course of ITP was determined following the international criteria [28], *i*.*e*. newly diagnosed ITP (less than 3 months of evolution), persistent ITP (3–12 months) and chronic ITP (more than 12 months). Differential diagnoses for ITP were ruled out in all patients. Bone marrow examination was performed in patients older than 60, or in case of systemic symptoms, or when anaemia or leukopenia were present, as recommended [1,2, 3]. The following biological parameters were collected: screening for glucose-6-phosphate dehydrogenase (G6PD) deficiency; the lowest platelet count within the 3 months prior to dapsone initiation, at response or after 4 weeks for non-responders, and at the time of the last response or the last follow-up; haemoglobin levels at dapsone initiation and at response, or after 4 weeks of treatments for non-responders.

### Definition of response

Response was defined as an increase in platelet count over 30x10^9^/L with at least a two-fold increase from the baseline in absence of bleedings, for at least 4 weeks. Partial response (PR) was considered when platelet count remain below 100x10^9^/L, and complete response (CR) when platelet count was over 100x10^9^/L. Non-response (NR) was considered when none of these criteria were achieved or when treatment was stopped because of side effects within the 4 weeks following its introduction. When another treatment (steroids or TPO-RA) was prescribed concomitantly with dapsone, only patients who had this treatment discontinued and maintained platelet counts over objectives were considered as responders. Patients lost to follow-up were considered as non-responders. Time to response was calculated from the day dapsone was initiated to the one of the first response was achieved. Duration of response was censored at the date of the last platelet count fulfilling response (PR or CR respectively) criteria.

### Statistics

Quantitative values are reported as median (1^st^-3^rd^ interquartile) and qualitative data as percentage. *P*-values were derived using Chi^2^ test and Mann-Whitney test for qualitative and quantitative values, respectively. Wilcoxon matched pairs test was used to compare platelet count before and after treatments. *P*<0.05 was considered significant. Statistical analyses were performed with STATA_TM_ Software (Stata Corporation).

## Results

### Patients

**Fig 1:**
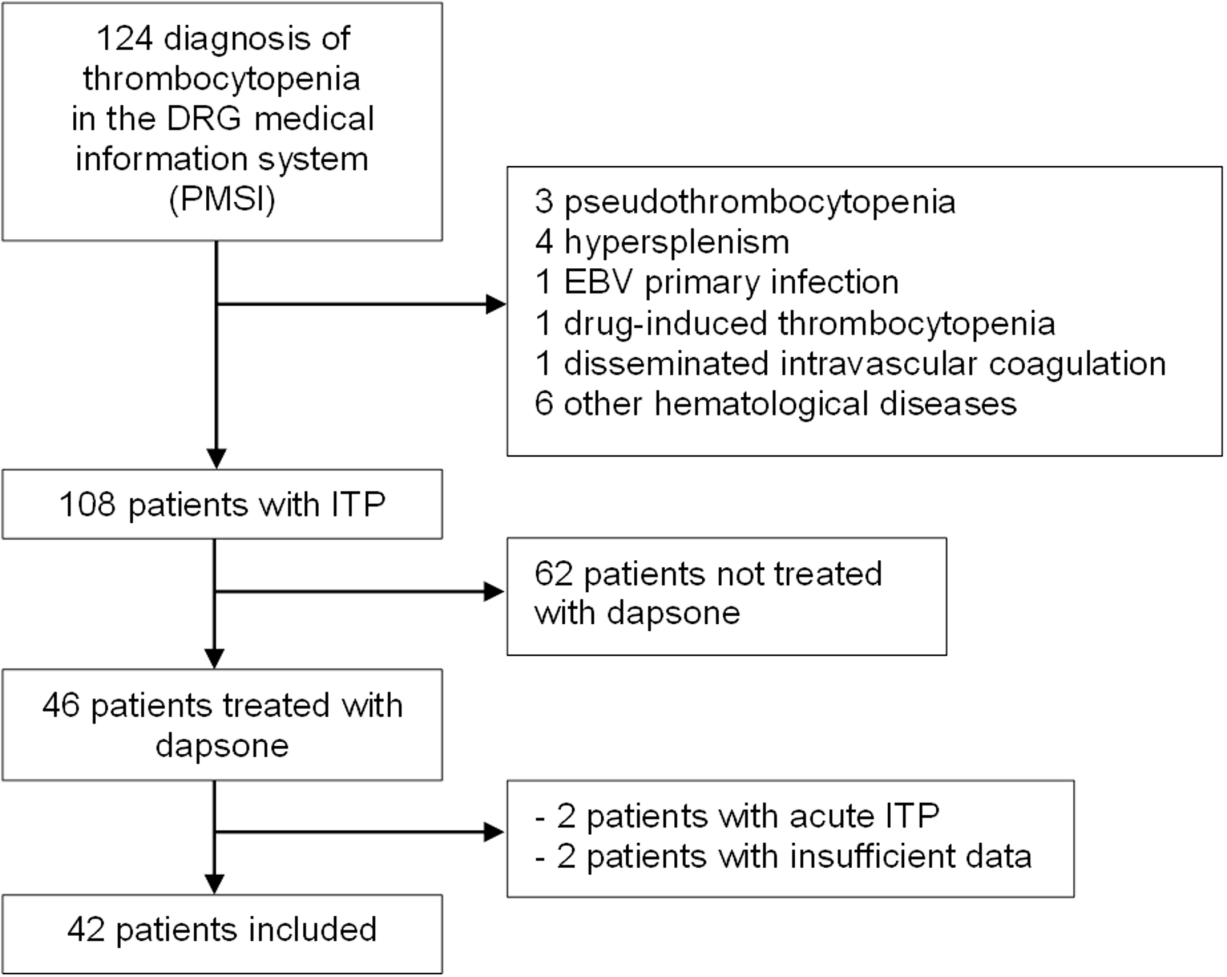
Flowchart of the study.

124 patients with thrombocytopenia were identified from our records between January 2006 and August 2016, among which 108 had ITP (Fig 1). Sixteen patients were excluded because of platelet aggregates (n = 3), hypersplenism (n = 4), Epstein-Barr virus infection (n = 1), drug-induced thrombocytopenia due to rifampicin (n = 1), disseminated intravascular coagulation (n = 1) or because thrombocytopenia was related to other hematologic diseases (myelodysplastic syndrome (n = 2), myeloma (n = 1), lymphoma (n = 1), chronic myelomonocytic leukaemia (n = 1), aplastic anaemia (n = 1)). Among these 108 ITP patients, 46 patients were treated with dapsone, but 4 patients were excluded because of acute ITP that resolved in less than 3 months (n = 2) or missing data (n = 2).

Clinical characteristics of patients are summarized in Table 1. Patients were 49.2 years old (31.2–68.2) at diagnosis. The median age at dapsone initiation was 57.1 (34.4–72.2), and 12 patients (28.6%) were older than 75. The female/male ratio was 1.8 (27/15). 14 patients (33.3%) had newly diagnosed ITP, 9 (21.4%) had persistent ITP and 19 (45.2%) had chronic ITP. Most of the patients had primary ITP (n = 38, 90.5%). Secondary ITP was associated with antiphospholipid syndrome (n = 2), sicca syndrome (n = 1) or Graves' disease (n = 1). Patients received a median of 1 (1–2) treatment before dapsone, consistent with steroids in all but one patient. For this patient, dapsone was used as first line therapy without steroids, because of type 2 diabetes associated with non-severe thrombocytopenia. For the others, prednisone was used at 1 mg/kg/d for a short duration (3 to 4 weeks) with an abrupt discontinuation. Seven patients did not respond to this first line therapy, while the others (34 out of 41 patients, 82.9%) responded but relapsed after discontinuation. IVIg were used in 16 patients (38.1%), among which 13/16 (81.3%) achieved a transient response. A TPO-RA was used in 5 patients (11.9%) before or concomitantly with dapsone: only one patient did not respond. Rituximab was used prior to dapsone in 7 patients (16.7%): 5 did not respond, 1 relapsed after 4 years, 1 had a PR and finally underwent splenectomy with a late relapse after 8 years. Hydroxychloroquine and vincristine were inefficient in 1 and 2 cases respectively.

**Table 1:** Clinical characteristics of patients treated with dapsone.

	Total	Responders	Non-responders	*p*-value
	n = 42	n = 23	n = 19	
Age, years	57.1 (34.4–77.2)	61.1 (26.9–81.3)	51.7 (37.7–67.5)	0.63
Sex (F/M)	27/15	16/7	11/8	0.43
Primary ITP	38 (90.5%)	21 (91.3%)	17 (89.5%)	1.00
Course of ITP, months	8.8 (2–53.6)	9.6 (1.6–49.9)	7.5 (2.3–57.9)	0.53
Newly diagnosed	14 (33.3%)	9 (39.1%)	5 (26.3%)	0.38
Persistent	9 (21.4%)	3 (13%)	6 (31.6%)	0.26
Chronic	19 (45.2%)	11 (47.8%)	8 (42.1%)	0.71
Treatments prior to dapsone, n	1 (1–2)	1 (1–2)	1 (1–3)	0.53
Steroids	41 (97.6%)	23 (100%)	18 (94.7%)	
IVIg	16 (38.1%)	7 (30.4%)	9 (47.4%)	
Rituximab	7 (16.7%)	2 (8.7%)	5 (26.3%)	
Splenectomy	1 (2.4%)	0 (0%)	1 (5.3%)	
TPO receptor agonist	5 (11.9%)	2 (8.7%)	3 (15.8%)	
Bleeding score at dapsone initiation	2 (0–5)	4 (1–5)	2 (0–5)	0.25
Haemoglobin level at dapsone initiation, g/dL	13.9 (12.9–14.9)	14 (13.3–15)	13.3 (12.9–14.8)	0.40
Nadir of platelet count within the 3 months prior dapsone initiation, x10^9^/L	14 (6–22)	12 (6–24)	14 (5–20)	0.97
Steroids at dapsone initiation	23 (54.8%)	14 (60.9%)	9 (47.4%)	0.38
Follow-up, months	67.6 (48.3–106.4)	64.3 (25.3–91.6)	76 (54.2–126.9)	0.14

The lowest median platelet count within the 3 months prior to dapsone initiation was 14x10^9^/L (6–22) and the bleeding score was 2 (0–5). Only 2 patients had a history of severe gastro-intestinal bleedings before dapsone initiation. Median follow-up was 67.6 months (49.5–105.2).

Dapsone was initiated at 100 mg/day for all but one patient, who had a CR with 100 mg 3 times a week. Dapsone was initiated in association with steroids in 54.8% (n = 23): prednisone was used at a median dose of 1 mg/kg/day and was discontinued after a median duration of 28 days (21–37.5). A TPO-RA was associated with dapsone in 4 cases: 2 did not respond to dapsone, 1 had a rash leading to dapsone withdrawal and the other maintained a CR after TPO-RA discontinuation.

### Response to dapsone

**Fig 2:**
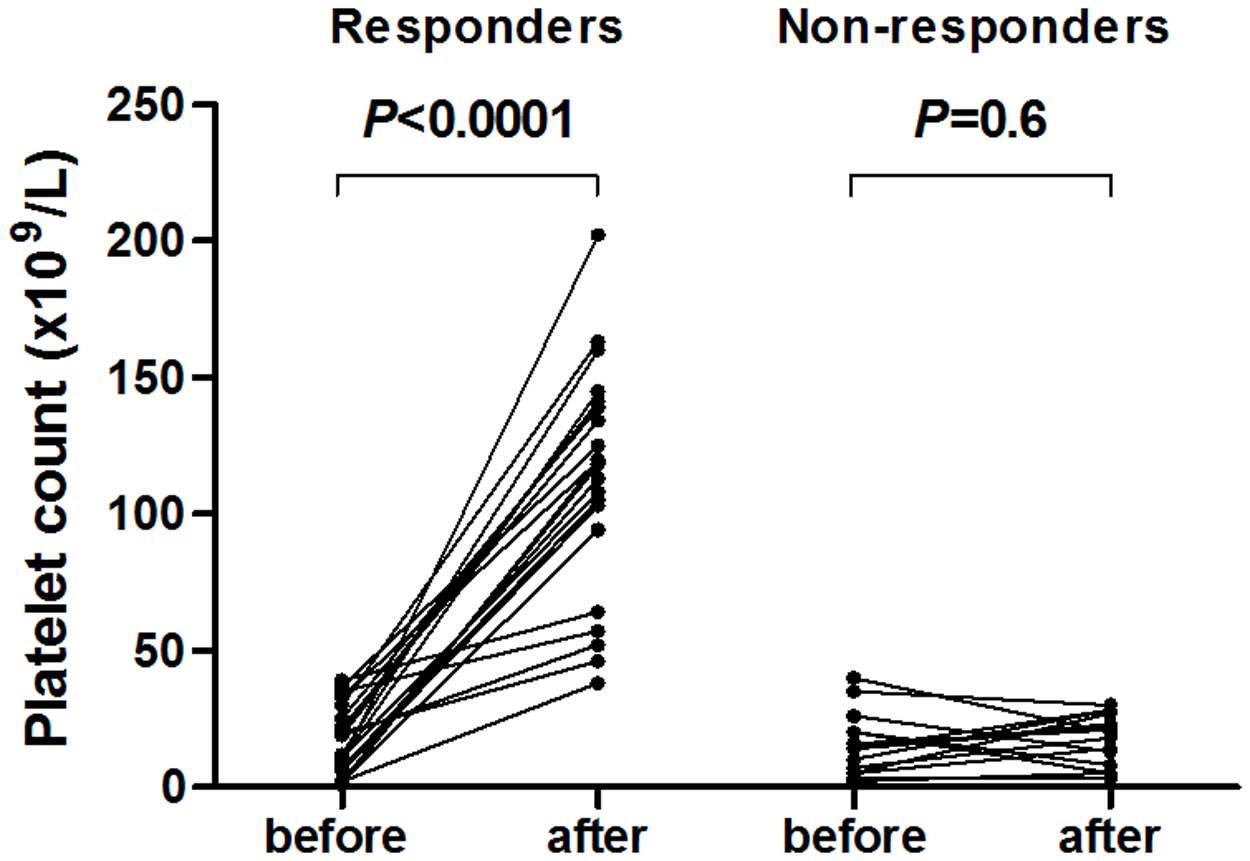
Platelet count before and after 4 weeks dapsone was started, in responders (n = 23) and non-responder patients (n = 19). *P*-value derived by Wilcoxon matched pairs test.

Response rates on dapsone are reported in Table 2 and platelet counts are depicted in Fig 2. Dapsone was given for a median duration of 8.9 (5.1–19.8) months for responders and 1.5 (0.6–2.4) months for non-responders. Median time to response was 29 (24–41) days, with 52.2% (12) patients responding in less than 1 month and only 3 after 2 months (60, 69 and 85 days). Platelet count significantly rose from 12x10^9^/L (6–25) to 119 (94–141) in responders (*p*<0.0001), but not in non-responders (14x10^9^/L (5–20) *vs*. 20 (8–27), *p* = 0.6; Fig 2). Overall response rate was 54.8% (n = 23), with CR in 38.1% (n = 16) and PR in 16.7% (n = 7). The median duration of response was 7.5 (3.5–19.4) months. Of note, 7 patients were considered as non-responders because of side effects. For the remaining 12 non-responders, dapsone was stopped after a median of 1.9 months (1.6–3). When responders and non-responders were compared, no significant difference was found concerning demographic factors or ITP history. Notably, the proportions of responders were not significantly different between newly diagnosed (n = 9/14, 64.2%), persistent (n = 3/9, 33.3%) and chronic ITP (n = 11/19, 57.9%). However, patients with a CR had a shorter ITP duration before dapsone initiation compared to patients with PR (2.2 (1.2–18.7) *vs*. 49.9 (38.5–90.9) months, *p* = 0.009). Moreover, response duration was longer in case of CR (10.4 (4.2–21.3) *vs*. 3.9 (1.9–7.1) months; *p* = 0.05). Patients who received dapsone in association with steroids tended to have more CR than PR (75.0% *vs*. 28.6%, *p* = 0.066). Importantly, all patients who were treated with this combination interrupted steroids after a median duration of 28 days, and previously experienced a relapse after the discontinuation of a first course of steroids. Thus, the fact that the response attributed to dapsone was indeed due to steroids appeared unlikely. However, to rule out this hypothesis, the response rates of dapsone started as monotherapy were also considered: a response was achieved in 47.4% (n = 9/19 patients) with a CR in 21.1% (n = 4/19) and a PR in 26.3% (n = 5/19).

**Table 2:** Outcome on dapsone therapy.

	All patients	Non-responders	Responders	Complete response	Partial response	*p*-value *
	(n = 42)	(n = 19)	(n = 23)	(n = 16)	(n = 7)	
Course of ITP at dapsone initiation, months	8.8 (2–53.6)	7.5 (2.3–57.9)	9.6 (1.6–49.9)	2.2 (1.2–18.7)	49.9 (38.5–90.9)	**0.009**
Course of ITP at dapsone initiation						**0.03**
Newly diagnosed, n (%)	14	5	9	9	0	
Persistent, n (%)	9	6	3	2	1	
Chronic, n (%)	19	8	11	5	6	
Time to response, days	29 (24–41)	/	29 (24–41)	28.5 (24–44)	32 (23–41)	0.84
Response duration, months	7.5 (3.5–19.4)	/	7.5 (3.5–19.4)	10.4 (4.2–21.3)	3.9 (1.9–7.5)	**0.05**
Treatment duration, months	3.9 (1.6–9)	1.5 (0.6–2.4)	8.9 (5.1–19.8)	12.1 (5.2–23)	7.1 (2.5–8.9)	0.08
Relapse on dapsone therapy	5 (11.9%)	/	5 (21.7%)	2 (12.5%)	3 (42.9%)	
Time to relapse, months	8.1 (6.5–13.6)	/	8.1 (6.5–13.6)	33.7 (20.9–46.4)	6.5 (4.6–10.1)	
Number of patients with sustained response (> 6 months) after dapsone cessation	5 (11.9%)	/	5 (21.7%)	4 (25%)	1 (14.3%)	
Follow-up after dapsone cessation, months	21.1 (12.6–29.5)	/	21.1 (12.6–29.5)	21.1 (7.5–29.5)	12.6	
Side effects	13 (31%)	7 (36.8%)	6 (26.1%)	3 (18.8%)	3 (42.9%)	
Cutaneous toxicity	5	5	0	0	0	
Methemoglobinemia	4	2	2	1	1	
Withdrawal because of toxicity	9	7	2	0	2	
Dosage adaptation because of toxicity	4	/	4	3	1	
Haemoglobin level at dapsone initiation, g/dL	13.9 (12.9–14.9)	13.3 (12.9–14.8)	14 (13.3–15)	13.5 (12.8–14.2)	15 (13.9–15.8)	**0.02**
Decrease in haemoglobin level, g/dL	1.5 (0.6–2.1)	1 (0.4–2)	1.8 (0.9–2.5)	1.5 (0.6–2)	2.5 (2–2.9)	**0.02**

* Complete response versus partial response

Haemoglobin level at dapsone initiation was 13.9 (12.9–14.9) g/dL, with a decrease in 1.5 (0.6–2.1) g/dL at response time. There was no significant difference in the decrease in haemoglobin level between responders and non-responders (1 (0.4–2) *vs*.1.8 (0.9–2.5) g/dL, respectively; *p* = 0.16).

**Fig 3:**
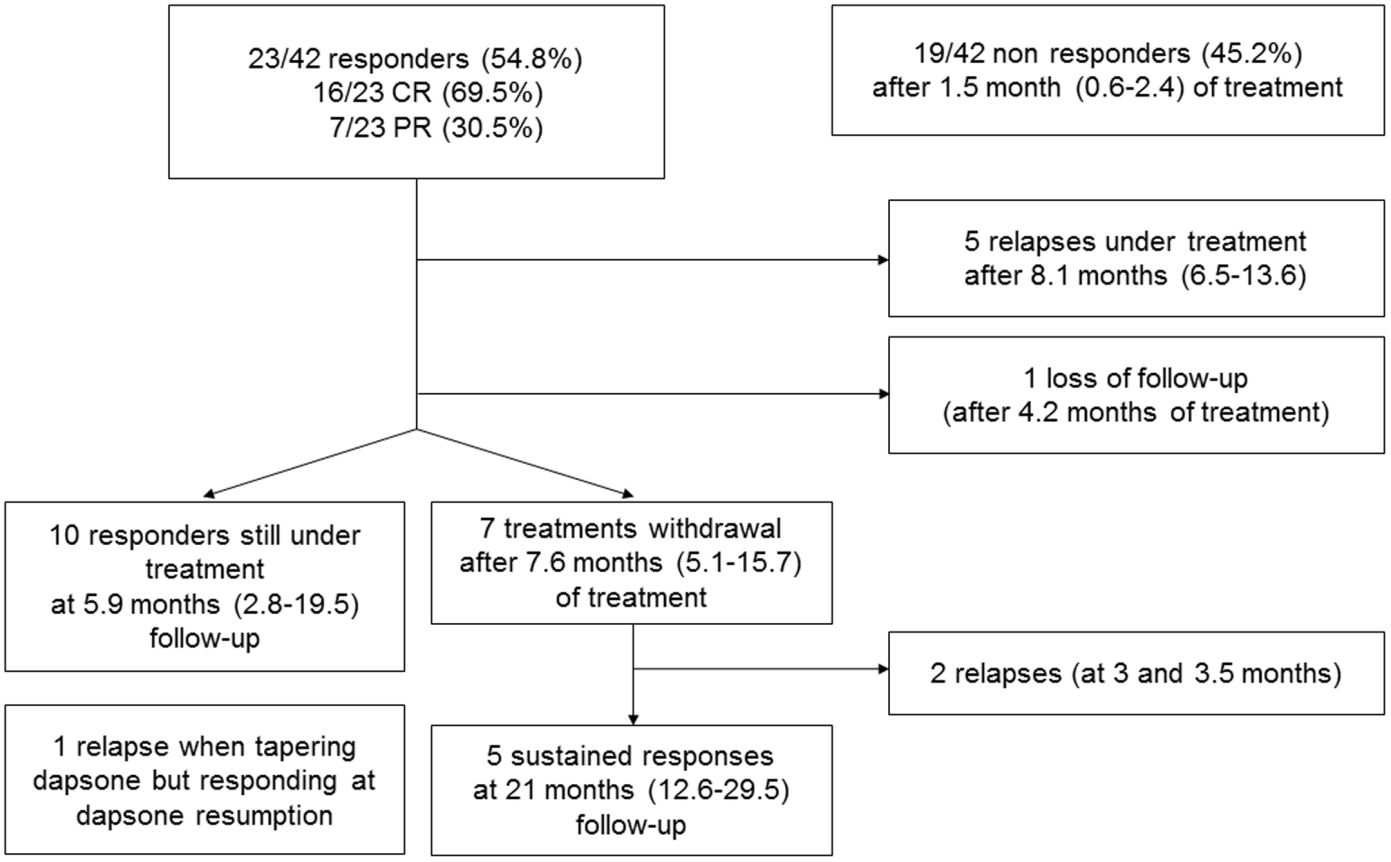
Flowchart of dapsone-treated patients. Follow-up is given by median (1^st^-3^rd^ interquartile).

Response to dapsone therapy is summarized in Fig 3. Among the 23 responders, a relapse occurred in 6 patients (26.2%). Five (21.7%) patients relapsed while on therapy, after 8.1 (6.5–13.6) months and received rituximab (n = 2) or underwent splenectomy (n = 2). One patient relapsed during dapsone tapering: a response was achieved after dapsone dosage was increased. Two other patients lost response at dapsone cessation but achieved response after dapsone was resumed.

Dapsone was definitively interrupted in 7 patients after 7.6 (5.1–15.7) months of response, after a progressive tapering for 3 patients. The reasons for dapsone cessation were side effects (n = 2) or desire of the patient to take off medication (n = 5). Two patients relapsed 3 and 3.5 months after dapsone cessation. Interestingly, a sustained response was observed in 5 patients (5/23 responders: 21.7%; 5/42 patients of the whole cohort: 14%) after a median follow-up of 21.1 (12.6–29.5) months. A CR was maintained in 3, while 2 switched to a PR. Among these 5 patients with sustained response after dapsone discontinuation, 3 had a newly diagnosed ITP and 2 a chronic ITP.

Ten patients were still responders under treatment after 5.9 (2.8–19.5) months. Dapsone dosage was tapered in 7 of them, in order to withdraw dapsone (n = 4) or because of side effects (n = 3). One patient was lost to follow-up after 4.2 months of response.

Regarding the 19 non-responders, a response was achieved with rituximab in 8/11, with splenectomy in 3/5, with TPO-RA in 3/4, and with hydroxychloroquine in 1/2, while 3 patients had a platelet count allowing a wait and watch attitude.

As expected, responders to dapsone required less second line treatments (30%, 7/23) compared to non-responders (84%, 16/19) after a median follow-up of 67.6 (49.5–105.2) months. Rituximab was used in 17% of responders compared to 58% of non-responders; splenectomy was performed in 17% *vs*. 26% and TPO-RAs were used in 4% *vs*.21%, respectively, supporting dapsone as an interesting medication to spare the use of expensive drugs or radical treatment such as splenectomy.

### Dapsone side-effects

Fifteen side effects were reported in 13 patients (31%), consistent with skin rash (n = 5), including sulfone syndrome (n = 2), methemoglobinemia (n = 4), neuropathy (n = 3), dyspnoea (n = 1), fatigue (n = 1) and diarrhoea (n = 1). Symptomatic anaemia or severe haemolysis was not observed. G6PD deficiency was screened in 36/42 patients (85.7%). There was no difference between patients with or without side effects, regarding age, ITP history, or the combination of steroids at dapsone initiation (Table 3). However, the proportion of women was higher in the group who experienced side effects (92.3% (12/13) *vs*. 51.7% (15/29), *p* = 0.015).

**Table 3:** Comparison of patients according to dapsone-related side effects.

	Side effects	No side effect	*p*-value
	(n = 13)	(n = 29)	
Age, years	56 (44.5–60.8)	62.7 (29.4–78.4)	0.87
Sex ratio (F/M)	12/1	15/14	**0.015**
Course of ITP, months	7.2 (1.9–29.3)	14.5 (2.1–62.7)	0.23
Primary / Secondary ITP	11/2	27/2	0.58
Newly diagnosed ITP, n	5 (38.5%)	9 (31%)	0.73
Persistent ITP, n	4 (30.8%)	5 (17.2%)	0.42
Chronic ITP, n	4 (30.8%)	15 (51.7%)	0.21
Number of treatments prior to dapsone, n	1 (1–2)	2 (1–2)	0.30
Steroid therapy associated to dapsone, n	8 (61.5%)	15 (51.7%)	0.56

Due to side effects, dapsone was withdrawn in 9 patients and the dosage reduced in 4, leading to the resolution of all the side effects, except for one case (neuropathy), for which dapsone accountability was not formally demonstrated. One patient died of pneumonia, which was not related to dapsone.

## Discussion

The characteristics of the population of this study are in accordance with previous reports on adult ITP with a median age of 57.1 years and a female predominance [29]. Dapsone was efficient in more than half of the patients (54.8%) with a CR achieved in more than one third (38.1%), irrespectively of the response to previous treatments and the course of ITP. These results are in line with literature, as summarized in Table 4, with a response rate ranging between 44.2% and 63.3%, and a CR in about 30% [20,22–26,30–34].

**Table 4:** Literature reports of dapsone in immune thrombocytopenia.

Study	Godeau *et al*.	Damodar *et al*.	Vancine *et al*.	Zaja *et al*.	Patel *et al*.^1^	Current study
Design	Prospective	Retrospective	Retrospective	Prospective	Retrospective	Retrospective
Number of patients (adults)	66	90 (55)	52 (45)	20 (20)	38 (26)	42 (42)
Age, *years*	48 (R)-43 (NR)	20.6 (3–61)	38 (13–78)	51 (27–74)	39.5 (20–68)	57.1 (34.4–77.2)
Course of ITP at dapsone introduction, *months*	52 (3–240)	24.5 (6–132)	5 (1–30)	46 (2–274)	-	8.8 (2–53.6)
Platelet count before dapsone, *x10*^*9*^*/L*	23 (2–49)	-	-	19	10 (1–21)	14 (6–22)
Average dose	75–100 mg/day	1–2 mg/kg/day	100 mg/day	100 mg/day	1.57mg/kg/day	33.3–100 mg/day
Overall response	33/66 (50%)^2^	57/90 (63.3%)^3^	23/52 (44.2%)^3^	11/20 (55%)^4^	12/26 (46.2%)^4^	23/42 (54.8%)^4^
Complete response	13/66 (19.7%)	44/90 (48.9%)	-	4/20 (20%)	10/26 (38.5%)	16/42 (38.1%)
Partial response	20/66 (30.3%)	13/90 (14.4%)	-	7/20 (35%)	2/26 (7.7%)	7/42 (16.7%)
Time to response, *days*	21 (8–90)	105 (30–270)	-	30 (15–60)	59 (27–108)	29 (24–41)
Response duration, *months*	-	-	-	42 (8–56)	14.2 (1.9–36.1)	7.5 (3.5–19.4)
Treatment duration in responders, *months*	-	9 (PR)-12.5 (CR)	9.7 (1–90.8)	31 (8–56)	5.3 (0.3–21.1)	8.9 (5.7–19.8)
Relapse on dapsone therapy	1/33 (3%)	-	-	0/11 (0%)	0/12 (0%) *2/6 children*	6/23 (26.1%)
Sustained response after discontinuation (> 6 months) in responder patients	1/33 (3%)	27/57 (47.4%)	15/23 (65.2%)	1/11 (9.1%)	2/12(16.7%)	5/23 (21.7%)
Side effects	16/66 (24.2%)	3/90 (3.3%)^5^	12/52 (23.1%)	2/20 (10%)	5/38 (13.2%)	13/42 (31%)
Withdrawal due to toxicity	7	3	2	0	2	9

^1^Study including adults and children, only adults are considered here

^2^PR defined as platelet count > 50x10^9^/L, CR as platelet count > 150x10^9^/L

^3^PR defined as platelet count > 50x10^9^/L, CR as platelet count > 100x10^9^/L

^4^PR defined as platelet count > 30x10^9^/L with at least a two-fold increase from baseline level, CR as platelet count > 100x10^9^/L

^5^Only severe side effects were reported

Relapse during treatment or dapsone tapering was observed in 22%, which is in accordance with previous reports [23,24,26]. Of note, a response can usually be achieved after starting again dapsone or by increasing its dosage.

More surprisingly, a sustained response after dapsone discontinuation was observed in 5 out of 7 responders who interrupted dapsone, representing around 21% of the responders. Interestingly, the response lasted more than 6 months in 5 patients, among which 2 had a chronic ITP. Such a sustained response ranged between 3 and 65.2% of the responders in previous reports [23,24,26], which is probably due to the difference in follow-up and the fact that dapsone discontinuation was not proposed to all responders, as most of the studies had a retrospective design. These results need to be confirmed in prospective studies and should be emphasized to the one reported for rituximab, 20% after 5 years [6], or TPO-RA, 15–30% [13–18]. Whether dapsone has immunomodulatory properties remains to be demonstrated, but these results show its potential interest to avoid or to delay radical treatment such as splenectomy. To date, mechanisms of action of dapsone in ITP remain unclear. It has been postulated that haemolysis triggered by dapsone could divert splenic macrophages, thus decreasing the phagocytosis of auto-antibody-coated platelet. This hypothesis was supported by a higher decrease in haemoglobin level in responders [23], which was not confirmed in our study and others [22,24,25,32].

The median time to response was 29 days, which is in line with previous reports (21–59 days) [23,24,26], except for one study that reported a longer time to response (105 days) [22]. In our study, late responses were observed in only 3 patients and occurred between the second and third months. Hence, for clinical practice, the efficacy of dapsone should be evaluated after 1 to 2 months, keeping in mind that late response is rare. Obviously, because of its long time to response, dapsone cannot be considered as an emergency treatment.

In this study, side effects related to dapsone were observed in 31%. Cutaneous effects were the most frequent, with sulfone syndrome, that is indeed a DRESS (Drug Rash with Eosinophilia and Systemic Symptoms), occurring only in 2 cases. It has been suggested that the concomitant prescription of steroids decreases the intensity of cutaneous side effects during ITP [35] compared to what is observed in other situations. This was not confirmed in our study, probably due to the small size of the cohort. Increase in methemoglobinemia level was observed in 10% of our patients but was symptomatic in only 1 case. Because of its low occurrence, methemoglobinemia can be checked only in symptomatic patients, keeping in mind that the transient interruption of dapsone or the decrease of its dosage usually allows resolution of symptoms. Of note, side effects led to dapsone discontinuation in 21% of our patients and to dapsone tapering in 9.5%, with a resolution in all but one case. Indeed, one case of neuropathy persisted after dapsone discontinuation but the accountability for dapsone was not firmly confirmed. Overall, the frequency of side effects reported here is higher than previously described, with ranges between 3 to 25% (Table 4) [22–26].

Unfortunately, no predictive factor of response to dapsone was identified. However, patients with ITP of short evolution had a higher rate of CR, which argues for the use of dapsone early in the course of ITP, notably for newly diagnosed or persistent ITP, which is in line with what was suggested in a previous report [26]. However, more than half of chronic ITP patients had a response to dapsone, supporting its use whatever the course of ITP. We did not confirm that a lower platelet count or a higher number of treatments received prior to dapsone were associated with non-response, contrary to what was previously reported [23,34].

In the past decade, new molecules such as rituximab and TPO-RA have improved the management of ITP. However, the cost of the long term use of these therapies can be very high and cheaper treatment options such as dapsone should be considered. The annual cost of dapsone is around 50 Euros, not considering biological exams, which should be compared to the one of TPO-RA that is around 26,000 Euros or to one course of intravenous rituximab (1000 mg, two weeks apart) that is approximatively 5,300 Euros, not considering hospitalization costs [10,36]. Even if medico-economic studies are lacking, dapsone appears as a cheaper option that should be tried whenever possible before using expensive drugs.

## Conclusions

Our results support the valuable place of dapsone as second line therapy in ITP with a response in about one half of the patients. Interestingly, a sustained response after its withdrawal can be observed, representing 5 out of our 23 responder patients. As expected, responders to dapsone will experience fewer treatments for ITP, thus decreasing the need for radical treatment such as splenectomy and for expensive therapies, which is of interest in the current economical context. However, side effects are frequent and reported in one quarter to one third of patients in retrospective studies. They are usually mild and resolved after dapsone tapering or discontinuation. Haemolysis is expected in all patients on treatment, and haemoglobin level should be checked during the first weeks of therapy and during any acute diseases that may precipitate anaemia, such as pulmonary diseases [26]. For this reason dapsone should be avoided in patients with severe pulmonary or cardiac diseases or with known haemolytic diseases. In that aim, screening for G6PD deficiency could be performed. Overall, prevention of major side effects mostly relies on patients’ education to recognize initial symptoms such as rash, fever, dyspnoea that require dapsone interruption and prompt medical assistance. Due to the time to response that is about one month, dapsone cannot be considered as an emergency therapy and its efficacy should not be assessed before one to two months. Of note, dapsone can be used in children [22,37] and can be maintained during pregnancy [38].

## Supporting information

S1 Table Anonymized data set. (XLS)
